# Trends in the place of death in Sweden from 2013 to 2019 – disclosing prerequisites for palliative care

**DOI:** 10.1177/26323524241238232

**Published:** 2024-03-16

**Authors:** Cecilia Larsdotter, Stina Nyblom, Hanna Gyllensten, Carl-Johan Furst, Anneli Ozanne, Ragnhild Hedman, Stefan Nilsson, Joakim Öhlén

**Affiliations:** Department of Nursing Science, Sophiahemmet University, Box 5605, Stockholm SE-114 86, Sweden; Institute of Medicine, Sahlgrenska Academy, University of Gothenburg, Gothenburg, Sweden; Palliative Centre, Sahlgrenska University Hospital, Gothenburg, Sweden; Institute of Health and Care Sciences, and Centre for Person-Centred Care (GPCC), Sahlgrenska Academy, University of Gothenburg, Gothenburg, Sweden; Faculty of Medicine, Department of Clinical Sciences Lund, The Institute for Palliative Care, Respiratory Medicine, Allergology, and Palliative Medicine, Lund University, Lund, Sweden; Institute of Health and Care Sciences, Sahlgrenska Academy, University of Gothenburg, Gothenburg, Sweden; Department of Neurology, Sahlgrenska University Hospital, Gothenburg, Sweden; Department of Nursing Science, Sophiahemmet University, Stockholm, Sweden; Institute of Health and Care Sciences, Sahlgrenska Academy, University of Gothenburg, Gothenburg, Sweden; Institute of Health and Care Sciences, and Centre for Person-centred Care (GPCC), Sahlgrenska Academy, University of Gothenburg, Gothenburg, Sweden; Palliative Centre, Sahlgrenska University Hospital, Gothenburg, Sweden

**Keywords:** end-of-life, palliative care, place of death, public health, trends

## Abstract

**Background:**

The drive for home care has increasingly impacted the organization and allocation of resources within the Swedish healthcare system.

**Objectives:**

With an interest in uncovering prerequisites for palliative care, this study aimed to investigate longitudinal trends in place of death within the adult Swedish population from 2013 to 2019 and examine potential associations between place of death and individual, geographic, and socioeconomic factors; hospital capacity; and healthcare utilization.

**Methods:**

This population-level comprehensive register study included all deceased individuals ⩾18 years old with a registered place of death (*n* = 599,137). Data were retrieved from public and patient data registers and the national register for palliative care. Trends and associations between place of death and co-variables were investigated by logistic regression- and interaction analyses.

**Results:**

From 2013 to 2019, the total number of home deaths increased by 1.9%, whereas the number of hospital deaths decreased by 2.6%. In the overall population of individuals living in their own homes, from 2013 to 2019, the likelihood of dying in hospital *versus* dying at home decreased (odds ratio: 0.98, 95% confidence interval: 0.97–0.99). Within the population with potential palliative needs living in their own home (78.4%), the likelihood of dying in hospitals equally decreased, except in Stockholm and the north region. For individuals residing in a nursing home, however, the likelihood of dying in hospital *versus* remaining in the nursing home until death only significantly decreased in the southern region.

**Conclusion:**

The results show a trend towards a decrease in hospital deaths but with cross-regional variations. Still, in 2019, only about one-fifth of all individuals died in their own homes. Public health-oriented interventions aimed at strengthening palliative care resources in nursing homes and home care are suggested.

## Introduction

Ageing societies with larger populations who live longer with long-term illness and enhanced needs for palliative care increasingly present healthcare systems with the challenge of organizing and providing timely and appropriate palliative care.^1^ It has been estimated that of all deceased individuals worldwide, approximately 74% have potential palliative care needs prior to death.^2^ A robust understanding of where people die is vital to support health policies, resource allocation, and service delivery in the planning and commissioning of palliative care services.^3^ Accordingly, over the past two decades both the place of death and dying in the individual’s preferred place have evolved into quality indicators and outcome measures for the state of palliative care in a country.^4,5^ Palliative care is a person-centred interdisciplinary approach to care, which aims to improve the quality of life and well-being of all people with serious illness and their family members. Central aspects of palliative care are early identification, assessment and treatment of symptoms and other problems; communication about end-of-life issues with patients and family members; shared decision-making; and support for family members.^6^ A palliative approach to care should, when needed, be applied in all care places,^7^ and can be provided on a general level at home, in nursing homes and in hospitals – through both specialized and non-specialized palliative care services (e.g. specialized palliative home care, hospital wards and hospices).^8^

Healthcare in Sweden is decentralized and regulated by the Health and Medical Service Act. Responsibility lies with the regional councils and municipal governments. Regional councils are overall responsible for organizing and delivering healthcare services, whereas municipalities for the most part are overall responsible for nursing homes and home care. Formally elected politicians in the regional councils are accountable to their citizens for the organization and distribution of equal and adequate healthcare services. Providers of healthcare, including care of older people, are either public or private, and with the same regulations applying to both. When regional councils buy services from private healthcare providers, it is based on a model where the healthcare is financed by the council but carried out by the private provider.^9^ Palliative care is integrated into the broader healthcare system and is provided at various levels of care, including in hospitals, nursing homes and home care services.

In societal discourses on severe illness and dying, there is a drive for home care, which has also increasingly impacted the organization and allocation of resources in most high-income countries.^6,10^ Furthermore, a recent review revealed that despite the higher consumption of outpatient resources, home care is – from a societal perspective – less costly than hospital care, especially over the last 2 months of life until death.^11^ In line with this, international studies show an overall preference among dying persons (and their family members) to be cared for and die in their own homes, provided that high-quality care and support to family members can be guaranteed.^12^

The place of death varies between countries and across patient groups. Although trends towards increased numbers of home deaths have been seen in some European countries, for example, United Kingdom,^13^ the opposite has also been seen with decreasing number of home deaths in countries such as Portugal,^14^ and overall, most people still die in hospitals and nursing homes.^15^ In 2012, in the first population-based place of death study in Sweden, we showed that 42.1% of all deaths occurred in hospitals and 38.1% in nursing homes, whereas only 17.8% of all deaths occurred in the person’s own home.^16^ Geographic and socioeconomic factors, as well as individual characteristics, are known to influence the place where people die.^16,17^ Moreover, cross-regional variations in places of death patterns were not explained by demographic differences or variations in the number of hospital and nursing home beds in the healthcare regions.

Traditionally, in place-of-death research, associations between individual, socioeconomic and geographic factors and place of death are analysed to understand place-of-death patterns. However, Gao *et al*.^18^ suggest that these factors alone do not explain these patterns. They have proposed a conceptual framework for evaluating the impact of health services’ infrastructure and organization on place of death, starting with palliative care policies informing and guiding the provision of palliative care, that is, types of care services, levels of palliative care, service capacity and geographical coverage. The focus when evaluating the impact of such policies, alongside the above-mentioned factors, is logically to examine and control for variables related to service utilization such as, for example, emergency department visits or specialist palliative care contacts. In Sweden, national clinical practice guidelines initiated by the profession, that is, physicians, nurses, etc.,^19^ and the first national guidance for palliative care on government initiative^10^ were launched in 2012 and implemented at the beginning of 2013, that is, following the above-mentioned 2012 study. The National Board of Health and Welfare (NBHW), in the national guidance for palliative care, stresses the importance of allocating resources and enhance palliative care competence so that peoples’ right to equal opportunities for palliative care, regardless of care place, can be maintained. Further, peoples’ right to make informed decisions about their care and care planning in partnership with ill persons and their families are articulated.^10^ If approximately half of all Swedish adult citizens, in line with the numbers in several other European countries, wish to be cared for and die in their own homes, a reasonable hypothesis is that the number of home deaths would have increased since the implementation of these policy documents. It is essential to follow-up the impact of these policy initiatives and the impact of the current structural changes in the provision of palliative care in society, and one way of doing this is by identifying longitudinal trends in place of death. Hence, with an interest in uncovering prerequisites for palliative care, we aimed to investigate longitudinal trends in place of death within the adult Swedish population from 2013 to 2019 and examine potential associations between place of death and individual, geographic, and socioeconomic factors, hospital capacity, and healthcare utilization including specialized palliative care.

## Design and methods

### Study population

This population-level comprehensive register study of longitudinal trends in place of death includes all deceased individuals ⩾18 years old in Sweden from 2013 to 2019, with a registered place of death. Death certificate data (sex, age, underlying cause of death and place of death) were obtained from the Swedish NBHW. Using the personal identity number of the deceased individuals, these death certificate data were linked with patient register data to obtain information regarding hospital transitions during the final month before death, and with the social service register to obtain information regarding nursing home residents’ time spent in nursing homes before death. Information about having received care in a specialist palliative care service was derived from the Swedish Register of Palliative Care (SRPC), while information regarding socioeconomic factors was obtained from public registers at Statistics Sweden (SCB). For details about the origin of the data from different registers, see Supplemental Table I. The numbers of hospital beds per 10,000 citizens in the healthcare regions were calculated based on open data from the Swedish Association of Local Authorities and Regions.

### Study variables

The primary outcome variable for all analyses was place of death, categorized into four distinct alternatives: hospital (unspecified speciality); home, that is, own private or rented home; nursing home, that is, including residential care settings and other forms of group dwellings; and other, for example, public places, roads, workplace. Inpatient palliative care services such as hospices and hospital-based palliative care beds or wards are not reported on the death certificates, and so these services are embedded in the hospital or nursing home categories. However, a dichotomous independent variable for having received specialized palliative care during the last week of life or not was derived and created from the SRPC information about having received care in a specialist palliative care service of any kind. Additionally, a variable was created for the subsample with potential palliative care needs according to the Murtagh *et al.*^20^ model, which is a refined approach for estimating palliative care needs within a population. The model is based on population-level death registration data, encompassing both underlying and contributory causes of death, and involves a more precise categorization of conditions relevant to palliative care, that is, aligning with international policy.^7^

Based on the framework by Gao *et al*.,^18^ variables for individual, geographic, socioeconomic characteristics and healthcare utilization known to affect place of death were included in the analyses. Underlying causes of death were grouped by International Statistical Classification of Diseases and Related Health problems (ICD-10) codes (see Supplemental Table I) into 11 categories: malignant neoplasms; diseases of the circulatory, digestive, nervous or respiratory system; endocrine/nutritional; infectious; mental and behavioural; dementia including senility diseases; external causes of morbidity; and other diseases. The ICD-10 code Z51.5 was also included, which is a medical classification for factors influencing health status and contact with health services that is supposed to be used by the physician who is responsible when the patient is eligible for palliative care. Other variables included were sex, age at death, year of death, geographic area of residence, degree of urbanization of the area of residence, healthcare region, number of hospital beds per 10,000/citizens, number of hospital transitions (transfers to or within hospital for care) during the last month before death, number of emergency visits during the last month before death, received specialized palliative care during the last week of life, time spent in nursing home before death (only relevant for nursing home residents), marital status, living conditions (number of adults in the household, number of children <18), educational attainment and birth country.

### Statistical analyses

For the investigation of the distribution of place of death and co-variables, percentages were calculated for each year and as a total. The analyses were performed separately, depending on whether the deceased individuals resided at home or in a nursing home at the time of death.

To investigate trends in the place of death, multivariable logistic regression analyses were performed for the total population. The dependent variable in the logistic regressions was place of death. The independent variable was year of death – adjusting for predefined individual, socioeconomic and environmental characteristics of the deceased. The predefined covariates used were birth country, living in a single-person household, number of children 18 years old or younger, marital status, educational attainment, received specialized palliative care during the last week of life, residing in an urban area, healthcare region, and number of hospital beds per 10,000 citizens per healthcare region. After this, the multivariable logistic regression analyses were repeated for the subpopulation with potential palliative care needs.

These analyses were then carried out for each of the six healthcare regions, using the same multivariable model with interaction of year of death for the subpopulation with potential palliative care needs. The models were performed twice, with year of death as linear and categorical variables, respectively. These analyses were stratified and performed separately, according to the living situation of the deceased, to understand the patterns of place of death, depending on whether people were living at home or in a nursing home (living at home and dying in hospital *versus* dying at home or in a nursing home, and living in a nursing home and dying in hospital *versus* dying in a nursing home. For calculations of the model with only nursing home residents, we only included the last four age categories (60+ years) in the logistic regression models to exclude disabled younger individuals who may have been living in a nursing home facility. Covariates were considered significantly associated with the outcome if *p* < 0.001.

Finally, to examine factors that could potentially be associated with the trend, that is, decrease in hospital deaths, interaction analysis was performed with calendar year, adjusted for all other variables. This was achieved by first taking the calendar year as the categorical variable with 2013 as reference. For each group or level of associated factor, the odds ratio (OR) was calculated for every year *versus* 2013. The analyses were performed with separate models for individuals residing at home but dying in hospital *versus* home as the dependent variable and for individuals residing in a nursing home but dying in hospital *versus* nursing home as the dependent variable, with individuals aged 60 years old and over. Probability Chi-square was considered significant if *p* < 0.001 to account for multiple comparisons. Area under Receiver Operating Characteristic (ROC)- curve (AUC-statistics) was calculated for description of goodness of predictors. All analyses were performed using SAS^®^ v9.4 (SAS-Institute, Cary, NC, USA).

The study followed the Strengthening the Reporting of Observational Studies in Epidemiology (STROBE) reporting guidelines.^21^ The STROBE checklist is provided as Supplemental File.

## Results

Of all 599,137 adult individuals (51.2% women; 48.8% men) who died in Sweden from 2013 to 2019, 40.7% died in hospital, 37.7% in a nursing home, 19.4% at home and the remaining 2.2% in other places. The most common underlying causes of death were circulatory diseases (35.0%) and neoplasms (26.6%). Only 10.0% had the ICD-diagnosis code for palliative care, whereas 78.4% were estimated to have potential palliative care needs.^19^. Of all individuals, 88.9% resided in an urban area at the time of death. The distribution of place of death and other variables for the total population are presented in Table 1. Cross-regional population characteristics are provided in Supplemental Table II.

**Table 1.** Distribution of place of death and other variables in the total adult death population from 2013 to 2019.

Variables	Total (*n* = 599,137)	Home 19.4%^a^ (*n* = 116,498)	Hospital 40.7% (*n* = 243,725)	Nursing home 37.7% (*n* = 225,849)	Other/unknown 2.2% (*n* = 13,065)
Deaths per year					
2013	83,750	15,532 (18.5%)	35,027 (41.8%)	31,497 (37.6%)	1694 (2.0%)
2014	83,053	15,497 (18.7%)	34,675 (41.8%)	31,226 (37.6%)	1655 (2.0%)
2015	85,440	16,480 (19.3%)	35,050 (41.0%)	32,178 (37.7%)	1732 (2.0%)
2016	86,158	17,079 (19.8%)	35,342 (41.0%)	31,860 (37.0%)	1877 (2.2%)
2017	87,799	17,191 (19.6%)	35,389 (40.3%)	33,302 (37.9%)	1917 (2.2%)
2018	88,033	17,386 (19.7%)	34,943 (39.7%)	33,555 (38.1%)	2149 (2.4%)
2019	84,904	17,333 (20.4%)	33,299 (39.2%)	32,231 (38.0%)	2041 (2.4%)
Sex					
Male	292,137	66,938 (22.9%)	128,514 (44.0%)	87,623 (30.0%)	9062 (3.1%)
Female	307,000	49,560 (16.1%)	115,211 (37.5%)	138,226 (45.0%)	4003 (1.3%)
Age at death					
18–29	4692	2032 (43.3%)	1262 (26.9%)	150 (3.2%)	1248 (26.6%)
30–39	4845	2206 (45.5%)	1525 (31.5%)	219 (4.5%)	895 (18.5%)
40–49	9755	3976 (40.8%)	4060 (41.6%)	558 (5.7%)	1161 (11.9%)
50–59	24,537	9164 (37.3%)	11,535 (47.0%)	2045 (8.3%)	1793 (7.3%)
60–69	65,355	21,099 (32.3%)	33,076 (50.6%)	8556 (13.1%)	2624 (4.0%)
70–79	130,665	30,968 (23.7%)	65,345 (50.0%)	31,471 (24.1%)	2881 (2.2%)
80–89	211,659	31,068 (14.7%)	85,649 (40.5%)	93,012 (43.9%)	1930 (0.9%)
90+	147,629	15,985 (10.8%)	41,273 (28.0%)	89,838 (60.9%)	533 (0.4%)
Underlying cause of death					
Malignant neoplasms	159,094	36,830 (23.1%)	77,190 (48.5%)	41,516 (26.1%)	3558 (2.2%)
Circulatory diseases	209,671	46,037 (22.0%)	78,738 (37.6%)	82,004 (39.1%)	2892 (1.4%)
Digestive diseases	19,146	2779 (14.5%)	13,355 (69.8%)	2894 (15.1%)	118 (0.6%)
Diseases of the nervous system	13,911	2021 (14.5%)	4995 (35.9%)	6812 (49.0%)	83 (0.6%)
Respiratory diseases	42,350	5618 (13.3%)	25,157 (59.4%)	11,366 (26.8%)	209 (0.5%)
Endocrine or nutritional diseases	17,512	4394 (25.1%)	5954 (34.0%)	7010 (40.0%)	154 (0.9%)
Infectious diseases	14,538	521 (3.6%)	10,555 (72.6%)	3437 (23.6%)	25 (0.2%)
Mental and behavioural diseases	3099	1043 (33.7%)	553 (17.8%)	1448 (46.7%)	55 (1.8%)
Dementia, including senility	65,478	2898 (4.4%)	4753 (7.3%)	57,756 (88.2%)	71 (0.1%)
External causes of morbidity	33,281	10,699 (32.1%)	12,961 (38.9%)	3930 (11.8%)	5691 (17.1%)
Other diseases	21,057	3658 (17.4%)	9514 (45.2%)	7676 (36.5%)	209 (1.0%)
Marital status^b^					
Married	193,947	40,936 (21.1%)	96,767 (49.9%)	51,849 (26.7%)	4395 (2.3%)
Unmarried	83,541	24,312 (29.1%)	31,673 (37.9%)	23,015 (27.5%)	4541 (5.4%)
Widow	222,925	28,507 (12.8%)	73,896 (33.1%)	118,895 (53.3%)	1627 (0.7%)
Divorced	98,465	22,646 (23.0%)	41,296 (41.9%)	32,070 (32.6%)	2453 (2.5%)
Educational attainment^b^					
No formal or elementary education	233,429	36,247 (15.5%)	87,909 (37.7%)	106,891 (45.8%)	2382 (1.0%)
Lower secondary education	52,189	12,600 (24.1%)	21,534 (41.3%)	16,032 (30.7%)	2023 (3.9%)
Higher secondary education	221,663	48,382 (21.8%)	94,239 (42.5%)	72,707 (32.8%)	6335 (2.9%)
Higher education	79,372	16,992 (21.4%)	34,886 (44.0%)	25,356 (31.9%)	2138 (2.7%)
Birth country					
Sweden	529,404	101,164 (19.1%)	212,490 (40.1%)	204,569 (38.6%)	11,181 (2.1%)
Outside Sweden	69,733	15,334 (22.0%)	31,235 (44.8%)	21,280 (30.5%)	1884 (2.7%)
Living situation^b^					
Home	447,628	107,994 (24.1%)	211,644 (47.3%)	116,118 (25.9%)	11,872 (2.7%)
Nursing home	109,683	3003 (2.7%)	19,606 (17.9%)	86,850 (79.2%)	224 (0.2%)
Other	12,260	2477 (20.2%)	4920 (40.1%)	4619 (37.7%)	244 (2.0%)
Residing in urban area^b^	532,435	99,206 (18.6%)	214,674 (40.3%)	207,647 (39.0%)	10,908 (2.0%)
Residing in rural area^b^	52,211	13,679 (26.2%)	21,996 (42.1%)	15,576 (29.8%)	960 (1.8%)
Healthcare region^b^					
North region	64,926	11,145 (17.2%)	24,280 (37.4%)	27,498 (42.4%)	2003 (3.1%)
Uppsala-Örebro region	136,991	26,757 (19.5%)	56,154 (41.0%)	51,704 (37.7%)	2376 (1.7%)
Stockholm region	107,992	19,033 (17.6%)	49,247 (45.6%)	36,333 (33.6%)	3379 (3.1%)
West region	109,165	20,821 (19.1%)	43,465 (39.8%)	42,755 (39.2%)	2124 (1.9%)
South-east region	69,483	15,198 (21.9%)	24,818 (35.7%)	28,478 (41.0%)	989 (1.4%)
South region	110,323	23,447 (21.3%)	45,668 (41.4%)	39,063 (35.4%)	2145 (1.9%)
Household situation					
Single-person household	322,180	57,966 (18.0%)	112,985 (35.1%)	146,197 (45.4%)	5032 (1.6%)
Multi-person household	274,388	57,931 (21.1%)	129,852 (47.3%)	78,862 (28.7%)	7743 (2.8%)
Children <18 in the household	18,118	4681 (25.8%)	7919 (43.7%)	4058 (22.4%)	1460 (8.1%)
Potential palliative care needs^c^	469,617	89,553 (19.1%)	181,251 (38.6%)	192,253 (40.9%)	6560 (1.4%)
No. of hospital transfers during the last month of life					
None	292,424	90,447 (30.9%)	25,734 (8.8%)	167,492 (57.3%)	8751 (3.0%)
One transfer	190,995	19,299 (10.1%)	124,901 (65.4%)	44,375 (23.2%)	2420 (1.3%)
Two or more transfers	115,718	6752 (5.8%)	93,090 (80.4%)	13,982 (12.1%)	1894 (1.6%)
No. of visits to emergency facilities during the last month of life					
None	400,877	97,033 (24.2%)	108,017 (26.9%)	184,989 (46.1%)	10,838 (2.7%)
One unplanned healthcare visit	152,003	14,405 (9.5%)	103,150 (67.9%)	32,768 (21.6%)	1680 (1.1%)
Two or more unplanned healthcare visits	46,257	5060 (10.9%)	32,558 (70.4%)	8092 (17.5%)	547 (1.2%)
Palliative care diagnosis; ICD-code Z51.5	59,878	13,731 (22.9%)	32,286 (53.9%)	11,642 (19.4%)	2219 (3.7%)
Cared for in specialized palliative care services at death^d^	66,281	21,474 (32.4%)	34,218 (51.6%)	7124 (10.7%)	3465 (5.2%)

a Percentages are row percentages.

b Missing data: Marital status *n* = 259; educational attainment *n* = 12,484; living situation *n* = 41,826; urban area (or not) *n* = 258; healthcare region n = 257; household situation *n* = 2569.

c Potential palliative care needs according to the Murtagh model.

d In- and outpatient specialized palliative or hospice care services, or specialized palliative home care services.

### Overall distribution and cross-regional variations in place of death

**Figure 1.**
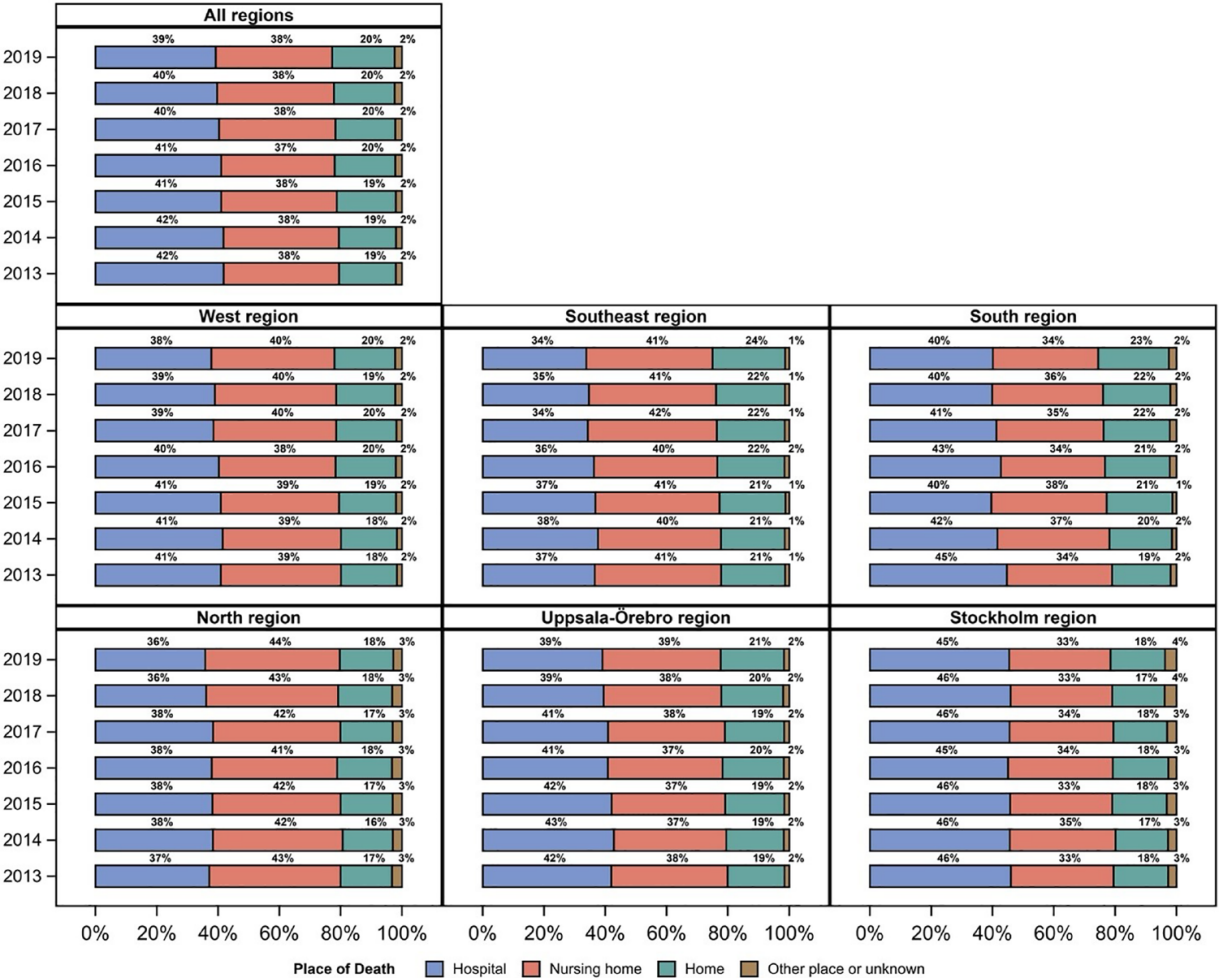
Overall and cross-regional distribution of place of death in Sweden per year (2013–2019).

From 2013 to 2019, the total number of home deaths in Sweden increased by 1.9%, whereas the number of hospital deaths decreased by 2.6%. The number of nursing home deaths only increased by 0.4%. The largest proportional regional increase in home deaths was seen in the south region (4.0%). This region also had the largest decrease in hospital deaths (4.6%). The smallest increase in home deaths was seen in Stockholm (0.1%). This was also the region with the smallest decrease in hospital deaths (0.6%). The distribution of overall and cross-regional home, hospital and nursing home deaths per year is presented in Figure 1.

### Trends in the place of death in the overall population

Within the total death population of individuals residing in their own home (*n* = 319,638), multivariable logistic regression analyses of the final models with year as the continuous variable showed a downward trend in the likelihood of dying in hospital *versus* dying at home [OR: 0.98, 95% confidence interval (CI): 0.97–0.99] from 2013 to 2019 (Supplemental Table III).

The separate analyses of individuals aged 60 years old or over showed that if residing in a nursing home (*n* = 105,522), the likelihood of dying in hospital *versus* dying in the nursing home decreased (OR: 0.98, 95% CI: 97–0.99), and the likelihood of those residing at home (*n* = 224,112) dying in the nursing home *versus* dying at home also decreased (OR: 0.98, 95% CI: 0.97–0.99) (Supplemental Table IV). However, the multivariable logistic regression analyses of the final models both for the total population with potential palliative care needs and for those aged 60 years old or over, with year as the categorical variable, show that the trend towards a decrease in hospital deaths was not consistent across all years (Supplemental Table V).

### Overall and cross-regional trends within the subpopulation with potential palliative care needs

**Figure 2.**
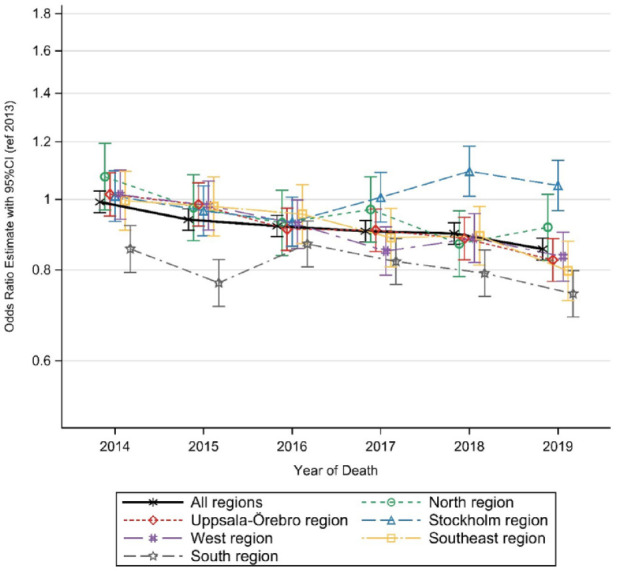
Living at home and dying in hospital *versus* dying at home among all individuals with potential palliative care needs from 2013 to 2019.

The results from the analyses (with year as a continuous variable) of the subpopulation with potential palliative care needs (78.4%) align with the overall population, with a decrease in the likelihood of dying in hospital *versus* dying at home among those living in their own home from 2013 to 2019. Cross-regional analyses showed a similar decrease in all regions except for Stockholm and the north region (Figure 2).

**Figure 3.**
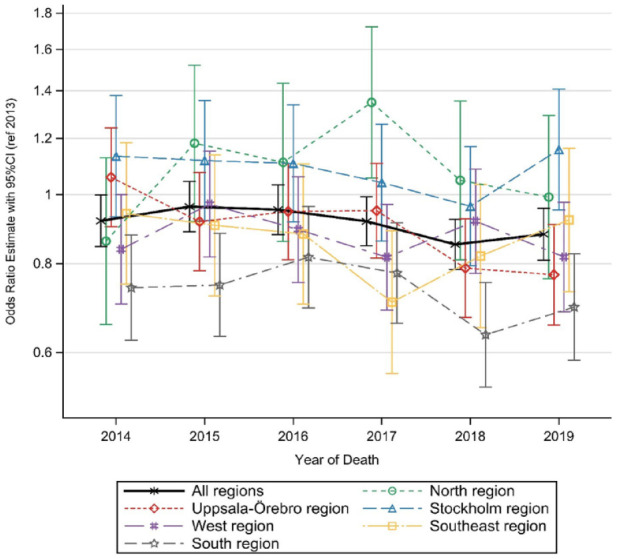
Living in nursing home and dying in hospital *versus* dying in nursing home among all individuals ⩾60 years with potential palliative care needs from 2013 to 2019.

For those aged 60 years old and over with potential palliative care needs and residing in a nursing home, the likelihood of dying in hospital *versus* remaining in the nursing home until their death only significantly decreased in the south region (Figure 3). Using year as a categorical variable in the analyses, the trends were not consistent for either of the groups across all the years (Supplemental Table VI).

### Interactions between trends in place of death of individuals with potential palliative care needs and associated variables

Besides healthcare region, factors that significantly interacted with the trends in place of death of individuals with potential palliative care needs residing in their own home were: being female (OR: 0.97, 95% CI: 0.97–0.98), being ⩾80 years old (OR: 0.97, 95% CI: 0.96–0.98), and not having received care in a specialized palliative care service during the last week of life (OR: 0.96, 95% CI: 0.95–0.97) or the ICD-10 code for palliative care (OR: 0.97, 95% CI: 0.96–0.98) for palliative care.

Regarding the older individuals residing in a nursing home and their likelihood of dying in hospital rather than residing in the nursing home until their death, there were no interactions between place of death and co-variables that particularly influenced the downward trend in hospital deaths.

In addition, we found that having received specialized palliative care (OR: 1.06, 95% CI: 1.05–1.08) at the time of death or having been diagnosed with palliative care (OR: 1.04, 95% CI: 1.03–1.05) significantly increased the odds of the younger population with potential palliative care needs to die in hospital rather than at home. Among the older individuals dwelling in nursing homes, the palliative care diagnosis also increased their likelihood of dying in hospital rather than in the nursing home (OR: 1.10, 95% CI: 1.05–1.17) (Supplemental Figures I–III).

## Discussion

The results from this study confirm a trend towards a decrease in hospital deaths. However, in 2019, still only around one-fifth of all deceased individuals died in their own homes. In the most recent study involving preferences for place of death in Sweden, 71% of a total general sample of approximately 2000 individuals stated that their own home was their preferred place to die.^22^ If most Swedish citizens share these and international preferences,^12^ which we can only assume is the case, there is a gap between preferences and place of death in Sweden.

We also found cross-regional variations. Stockholm and the north region did not follow the trend of a decrease in hospital deaths within the home-dwelling population with potential palliative care needs. For those aged 60 years old or over residing in a nursing home, the likelihood of dying in hospital *versus* remaining in the nursing home until death only significantly decreased in the southern region. These cross-regional variations were not statistically associated with number of hospital beds in the regions and, hence, it can be assumed that these variations are related to other infrastructural or organizational factors, such as existing variations in availability and types of specialized palliative care services in the different healthcare regions.^23^

Six years of national palliative care policy implementation may be a somewhat short period of time to expect a considerable change in the place of death of a total population. The speed of change or effect after launching a policy may vary depending on several factors, such as the complexity and specificity of the policy, the resources available for implementation and the level of support from stakeholders and the public.^24^ As far back as 1997, the Swedish national prioritization proposition, guided by ethical principles and general guidelines on priority setting in healthcare, established that end-of-life and palliative care should be attended to in the highest priority group within the entire healthcare system.^25^ The national guidance for palliative care^10^ stresses the need for equity in availability and access to palliative care across the country, and at the same time point to the challenge with the decentralized structure for healthcare responsibility. The document, however, lacks clear steering directives or strategies.

One factor that may contribute to the gap between political will and resource allocation is the complexity of healthcare systems. In a study by Centeno *et al*.^1^ about barriers to implementation of palliative care in European countries, Sweden was among the countries in which a lack of equal regulations due to healthcare regions’ autonomous governance and organizing of health and social care, as well as a lack of coordination within the healthcare system, were flagged as main policy barriers. Furthermore, the latest NBHW report about the adherence to guidelines for palliative care in Sweden^26^ stresses the significant differences in palliative care provision in regional councils and municipalities and the major disparities related to staff training and instruction, symptom identification/treatments, end-of-life conversations, care planning, family member support, etc., all of which are important quality aspects of palliative care.

Another main challenge to healthcare policy implementation is resistance from healthcare providers and institutions. In addition, policies that are not well-monitored can be easily ignored or undermined, leading to non-compliance and ineffective implementation.^24^

Interestingly, secondary findings showed a significant interaction between hospital deaths and having been diagnosed with the palliative care diagnosis within the total population with potential palliative needs, and for those who were home dwellers also when specialized palliative care had been received in the last week of life. The NBHW, in their death certificates, only allow a choice between four places of death categories. Consequently, deaths occurring in specialized palliative care services provided by the regions are classified as hospital deaths, and deaths occurring in a hospice provided by municipalities are classified as nursing home deaths. This may be part of the explanation, and, hence, further inquiry into specialized palliative care services is needed.

The interaction between increased likelihood of hospital death if residing in a nursing home and receiving the palliative care diagnosis, however, is even more concerning as this suggests insufficiency in the governance, organization, resources or competencies (or all four) regarding the provision of palliative care in nursing homes, resulting in late-stage hospital transfers. The COVID pandemic highlighted that clinical routines for palliative care are not in place in Swedish nursing homes,^26^ which has also been recognized in previous research,^27,28^ although some good examples have been brought to the fore.^29^ Initiatives have been taken on a national level to strengthen the general nursing home staff competence and coordination of care between care providers as parts of national good quality local care reform. In brief, this reform entails allocating enhanced resources and responsibility for care to the regions and municipalities. Directives appointing end-of-life and palliative care within this reform, however, are vaguely articulated despite being a prioritized area.

The present study has methodological limitations; as was previously mentioned, the four options for places of death on the death certificates exclude the possibility of population-level identification of individuals who died in either specialized palliative care services or hospices that are provided by a municipality. This means that these individuals are included in the number of hospital or nursing home deaths. Furthermore, there is no exact information available in Sweden about cross-regional capacity of specialized inpatient or home palliative care services, primary care, or nursing homes, and, hence, this could not be calculated in relation to its potential associations with place of death. Hence, the conceptual framework suggested for place-of-death studies by Gao *et al*.^18^ requires more developed healthcare data related to care at the end-of-life on national level than what is available in Sweden today.

## Conclusion and implications

The results from this study confirm a trend towards a decrease in hospital deaths in Sweden from 2013 to 2019 but with cross-regional variations and inconsistencies. That is, the results also show that having received specialized palliative care or having been diagnosed with palliative care significantly increased the odds of the younger population with potential palliative care needs to die in hospital rather than at home. These findings may be methodological due to data limitations related to place of death but require further attention and in-depth understanding. Furthermore, for those residing in nursing homes, the palliative care diagnosis increased the odds of dying in hospital, which suggests insufficiency regarding the provision of palliative care in nursing homes.

Still, this study clearly shows that in 2019, only around one-fifth of all individuals died in their own homes. The impact of a policy change was assumed in designing this study. However, it could be argued that 7 years is too short a period for a society to accomplish transformation of care structures and allocation of resources and competence, and further changes may have taken place over the past 3 years. Nevertheless, these results highlight the urgent need to prioritize, on the political healthcare agenda, the existing challenge in organizing and implementing palliative care in the healthcare regions in a way that promotes equal access to adequate care for all groups within the whole country while respecting people’s preferences regarding place of end-of-life care and death. The results raise questions about how decision-makers with responsibility for macro healthcare decision-making reason about strategies that would promote equity in palliative care. Public health-oriented interventions are suggested focusing on strengthening palliative care resources in nursing homes and home care, for example, structured knowledge implementation or implementation of palliative care consultation teams, which has been shown to potentially drive a palliative orientation in patient care that enables care according to people’s preferences.^30^

## Supplemental Material

sj-docx-1-pcr-10.1177_26323524241238232 – Supplemental material for Trends in the place of death in Sweden from 2013 to 2019 – disclosing prerequisites for palliative care Supplemental material, sj-docx-1-pcr-10.1177_26323524241238232 for Trends in the place of death in Sweden from 2013 to 2019 – disclosing prerequisites for palliative care by Cecilia Larsdotter, Stina Nyblom, Hanna Gyllensten, Carl-Johan Furst, Anneli Ozanne, Ragnhild Hedman, Stefan Nilsson and Joakim Öhlén in Palliative Care and Social Practice

sj-docx-2-pcr-10.1177_26323524241238232 – Supplemental material for Trends in the place of death in Sweden from 2013 to 2019 – disclosing prerequisites for palliative care Supplemental material, sj-docx-2-pcr-10.1177_26323524241238232 for Trends in the place of death in Sweden from 2013 to 2019 – disclosing prerequisites for palliative care by Cecilia Larsdotter, Stina Nyblom, Hanna Gyllensten, Carl-Johan Furst, Anneli Ozanne, Ragnhild Hedman, Stefan Nilsson and Joakim Öhlén in Palliative Care and Social Practice

sj-docx-3-pcr-10.1177_26323524241238232 – Supplemental material for Trends in the place of death in Sweden from 2013 to 2019 – disclosing prerequisites for palliative care Supplemental material, sj-docx-3-pcr-10.1177_26323524241238232 for Trends in the place of death in Sweden from 2013 to 2019 – disclosing prerequisites for palliative care by Cecilia Larsdotter, Stina Nyblom, Hanna Gyllensten, Carl-Johan Furst, Anneli Ozanne, Ragnhild Hedman, Stefan Nilsson and Joakim Öhlén in Palliative Care and Social Practice

## Declarations

**Ethics approval and consent to participate:** The Swedish Ethical Review Authority stated that the study could be conducted without ethical review because the sample consists of deceased individuals (no. 2019-05213, 2020-01758).

**Consent for publication:** Not applicable.

**Availability of data and materials:** The data for this study are available from each register holder. Certain restrictions may apply.
