# Supplementary material for: Trends in the place of death in Sweden from 2013 to 2019 – disclosing prerequisites for palliative care
Source: Palliat Care Soc Pract. 2024 Mar 16;18:26323524241238232. doi: 10.1177/26323524241238232 (PMC10943753; doi:10.1177/26323524241238232)
Supplement: sj-docx-1-pcr-10.1177_26323524241238232 – Supplemental material for Trends in the place of death in Sweden from 2013 to 2019 – disclosing prerequisites for palliative care [file sj-docx-1-pcr-10.1177_26323524241238232.docx]

**Supplementary Tables I-VII**

Supplementary Table I. List of variables and registers.

Supplementary Table II. Cross-regional population characteristics.

Supplementary Table III. Multivariable regression analysis for residing at home and dying in hospital vs. dying at home (whole population)

Supplementary Table IV. Multivariable logistic regression analysis for residing in a nursing home and dying in hospital vs. dying in a nursing home for those aged 60 years old and over (whole population).

Supplementary Table V. Multivariable logistic regression analysis for residing at home and dying in a nursing home versus dying at home (whole population)

Supplementary Table VI. Odds ratios from multivariable logistic regression analyses for trends in place of death of people with potential palliative care needs.

**Supplementary Figures I-III**

Supplementary Figure I. Interaction of year of death with having received specialised palliative care during the last week of life for those living at home.

Supplementary Figure II. Interaction of year of death with having been diagnosed with ICD-10 code Z51.5 for palliative care for those living at home.

Supplementary Figure III. Interaction of year of death with having been diagnosed with ICD-10 code Z51.5 for palliative care for those living in a nursing home.
