# Supplementary material for: Trends in the place of death in Sweden from 2013 to 2019 – disclosing prerequisites for palliative care
Source: Palliat Care Soc Pract. 2024 Mar 16;18:26323524241238232. doi: 10.1177/26323524241238232 (PMC10943753; doi:10.1177/26323524241238232)
Supplement: sj-docx-2-pcr-10.1177_26323524241238232 – Supplemental material for Trends in the place of death in Sweden from 2013 to 2019 – disclosing prerequisites for palliative care [file sj-docx-2-pcr-10.1177_26323524241238232.docx]

Supplementary Table I. List of variables and registers

| **Variables** | **Categorization** | **Register** |
| --- | --- | --- |
| Social identification number |  | Death certificate register NBHW |
| Sex | Male, female, other | Death certificate register NBHW |
| Age | 0–18, 19–49, 41–69, 70–89, 90+ | Death certificate register NBHW |
| Underlying cause of death |  | Death certificate register NBHW |
| Types of cancer |  | Death certificate register NBHW |
| Palliative care diagnosis | ICD-10 code Z 51.5 | Death certificate register NBHW |
| Place of death | Home, hospital, nursing home, other | Death certificate register NBHW |
| No. of hospital transitions during the last month before death | String | Patient data register NBHW |
| No. of emergency visits during the last month of life | String | Patient data register NBHW |
| Time spent in nursing home (only nursing home residents) before death | String | Social service register NBHW |
| Cared for in specialised palliative care services at death | Yes/No | Swedish Register for Palliative Care |
| Marital status | Unmarried, married, widowed or divorced | Mona, SCB |
| Living conditions | No. of adults 18 or older, and no. of children <18 in the household | Mona, SCB |
| Birth country | Categorized in regions according to SCB | Multi-generation register, SCB |
| Parents’ birth country | Categorized in regions according to SCB | Multi-generation register, SCB |
| Educational attainment | Operationalized according to the Swedish classification system SUN2000 into “no formal or elementary education”, “lower secondary education”, “higher secondary education” and “higher education | Mona, SCB |
| Geographic area of residence | Municipality | Mona, SCB |
| Degree of urbanisation of the area of residence | Categorisation made by SCB, defining urban as an area with continuous settlements/houses, with 200 metres or less between the houses and with at least 200 citizens | Mona, SCB |
| Health care region | Based on the six existing healthcare regions | Mona, SCB |
| Hospital capacity | Number of hospital beds per 10,000 citizens in each of the healthcare regions | Statistics from NBHW |
| Nursing home capacity | Number of nursing home beds per 10,000 citizens in each of the healthcare regions | Statistics from NBHW |
| Specialised palliative care capacity | In-patient beds and home care capacity in services for specialised palliative care per 10,000 citizens in each of the healthcare regions | Swedish Register for Palliative Care, and when needed, direct contact with Leads of regional palliative care processes and services |

Supplementary Table II. Cross-regional population characteristics

| **Variable** |  | | | | | | |
| --- | --- | --- | --- | --- | --- | --- | --- |
|  | **Total (n=598880)** | **North region (n=64926)** | **Uppsala-Örebro region (n=136991)** | **Stockholm region (n=107992)** | **West region (n=109165)** | **Southeast region (n=69483)** | **South region (n=110323)** |
| **Sex** |  |  |  |  |  |  |  |
| **Male** | 291931 (48.7%) | 31976 (49.2%) | 67412 (49.2%) | 51370 (47.6%) | 53606 (49.1%) | 33883 (48.8%) | 53684 (48.7%) |
| **Female** | 306949 (51.3%) | 32950 (50.8%) | 69579 (50.8%) | 56622 (52.4%) | 55559 (50.9%) | 35600 (51.2%) | 56639 (51.3%) |
| **Age at death** |  |  |  |  |  |  |  |
| **90+** | 147621 (24.6%) | 13971 (21.5%) | 32764 (23.9%) | 27461 (25.4%) | 27318 (25.0%) | 18044 (26.0%) | 28063 (25.4%) |
| **18–29** | 4680 (0.8%) | 553 (0.9%) | 1069 (0.8%) | 958 (0.9%) | 920 (0.8%) | 446 (0.6%) | 734 (0.7%) |
| **30–39** | 4829 (0.8%) | 457 (0.7%) | 1073 (0.8%) | 1070 (1.0%) | 901 (0.8%) | 477 (0.7%) | 851 (0.8%) |
| **40–49** | 9728 (1.6%) | 873 (1.3%) | 2087 (1.5%) | 2189 (2.0%) | 1832 (1.7%) | 1060 (1.5%) | 1687 (1.5%) |
| **50–59** | 24490 (4.1%) | 2413 (3.7%) | 5302 (3.9%) | 5055 (4.7%) | 4661 (4.3%) | 2653 (3.8%) | 4406 (4.0%) |
| **60–69** | 65287 (10.9%) | 6883 (10.6%) | 15099 (11.0%) | 12542 (11.6%) | 11820 (10.8%) | 7006 (10.1%) | 11937 (10.8%) |
| **70–79** | 130608 (21.8%) | 14637 (22.5%) | 30664 (22.4%) | 23823 (22.1%) | 23047 (21.1%) | 14811 (21.3%) | 23626 (21.4%) |
| **80–89** | 211637 (35.3%) | 25139 (38.7%) | 48933 (35.7%) | 34894 (32.3%) | 38666 (35.4%) | 24986 (36.0%) | 39019 (35.4%) |
| **Underlying cause of death** |  |  |  |  |  |  |  |
| **Neoplasms** | 159053 (26.6%) | 15778 (24.3%) | 36272 (26.5%) | 29618 (27.4%) | 28875 (26.5%) | 18261 (26.3%) | 30249 (27.4%) |
| **Diseases of the circulatory system** | 209591 (35.0%) | 22474 (34.6%) | 49000 (35.8%) | 34821 (32.2%) | 38807 (35.5%) | 25955 (37.4%) | 38534 (34.9%) |
| **Diseases of the digestive system** | 19132 (3.2%) | 2053 (3.2%) | 4544 (3.3%) | 3487 (3.2%) | 3348 (3.1%) | 2108 (3.0%) | 3592 (3.3%) |
| **Diseases of the nervous system** | 13908 (2.3%) | 1629 (2.5%) | 3001 (2.2%) | 2722 (2.5%) | 2491 (2.3%) | 1551 (2.2%) | 2514 (2.3%) |
| **Diseases of the respiratory system** | 42341 (7.1%) | 4396 (6.8%) | 9474 (6.9%) | 7446 (6.9%) | 7842 (7.2%) | 4853 (7.0%) | 8330 (7.6%) |
| **Endocrine and nutritional diseases** | 17508 (2.9%) | 1911 (2.9%) | 4026 (2.9%) | 2616 (2.4%) | 3459 (3.2%) | 2138 (3.1%) | 3358 (3.0%) |
| **Infectious diseases** | 14535 (2.4%) | 1566 (2.4%) | 3403 (2.5%) | 2322 (2.2%) | 2778 (2.5%) | 1658 (2.4%) | 2808 (2.5%) |
| **Mental and behavioural diseases** | 3095 (0.5%) | 345 (0.5%) | 599 (0.4%) | 472 (0.4%) | 801 (0.7%) | 308 (0.4%) | 570 (0.5%) |
| **Dementia, including senility** | 65473 (10.9%) | 8884 (13.7%) | 14522 (10.6%) | 13951 (12.9%) | 10959 (10.0%) | 6695 (9.6%) | 10462 (9.5%) |
| **External causes of morbidity** | 33206 (5.5%) | 3831 (5.9%) | 7609 (5.6%) | 6430 (6.0%) | 6017 (5.5%) | 3631 (5.2%) | 5688 (5.2%) |
| **Other** | 21038 (3.5%) | 2059 (3.2%) | 4541 (3.3%) | 4107 (3.8%) | 3788 (3.5%) | 2325 (3.3%) | 4218 (3.8%) |
| **Living conditions, Number of children aged under 18 years old** |  |  |  |  |  |  |  |
| **No children under 18** | 578450 (97.0%) | 63112 (97.3%) | 132960 (97.3%) | 102758 (95.9%) | 105351 (96.8%) | 67522 (97.4%) | 106747 (97.2%) |
| **Children under 18** | 18118 (3.0%) | 1724 (2.7%) | 3656 (2.7%) | 4400 (4.1%) | 3456 (3.2%) | 1799 (2.6%) | 3083 (2.8%) |
| **Living in single-person household** |  |  |  |  |  |  |  |
| **Single-person household** | 322180 (54.0%) | 34597 (53.4%) | 74044 (54.2%) | 57593 (53.7%) | 58239 (53.5%) | 37791 (54.5%) | 59916 (54.6%) |
| **Multi-person household** | 274388 (46.0%) | 30239 (46.6%) | 62572 (45.8%) | 49565 (46.3%) | 50568 (46.5%) | 31530 (45.5%) | 49914 (45.4%) |
| **Potential palliative care needs** |  |  |  |  |  |  |  |
| **NO** | 129402 (21.6%) | 14294 (22.0%) | 29643 (21.6%) | 22907 (21.2%) | 23763 (21.8%) | 14700 (21.2%) | 24095 (21.8%) |
| **Potential palliative care needs** | 469478 (78.4%) | 50632 (78.0%) | 107348 (78.4%) | 85085 (78.8%) | 85402 (78.2%) | 54783 (78.8%) | 86228 (78.2%) |
| **Year of Death** |  |  |  |  |  |  |  |
| **2013** | 83703 (14.0%) | 9080 (14.0%) | 19080 (13.9%) | 14715 (13.6%) | 15256 (14.0%) | 9994 (14.4%) | 15578 (14.1%) |
| **2014** | 83005 (13.9%) | 8978 (13.8%) | 18609 (13.6%) | 15132 (14.0%) | 15008 (13.7%) | 9806 (14.1%) | 15472 (14.0%) |
| **2015** | 85390 (14.3%) | 9219 (14.2%) | 19601 (14.3%) | 15562 (14.4%) | 15438 (14.1%) | 9917 (14.3%) | 15653 (14.2%) |
| **2016** | 86122 (14.4%) | 9291 (14.3%) | 19928 (14.5%) | 15622 (14.5%) | 15606 (14.3%) | 9887 (14.2%) | 15788 (14.3%) |
| **2017** | 87743 (14.7%) | 9533 (14.7%) | 20069 (14.6%) | 15685 (14.5%) | 16078 (14.7%) | 10261 (14.8%) | 16117 (14.6%) |
| **2018** | 88032 (14.7%) | 9498 (14.6%) | 20156 (14.7%) | 16035 (14.8%) | 16127 (14.8%) | 10136 (14.6%) | 16080 (14.6%) |
| **2019** | 84885 (14.2%) | 9327 (14.4%) | 19548 (14.3%) | 15241 (14.1%) | 15652 (14.3%) | 9482 (13.6%) | 15635 (14.2%) |
| **Marital status** |  |  |  |  |  |  |  |
| **Married** | 193947 (32.4%) | 20520 (31.6%) | 44026 (32.1%) | 33428 (31.0%) | 36002 (33.0%) | 23329 (33.6%) | 36642 (33.2%) |
| **Unmarried** | 83541 (13.9%) | 10227 (15.8%) | 19145 (14.0%) | 16425 (15.2%) | 15022 (13.8%) | 8892 (12.8%) | 13830 (12.5%) |
| **Widow** | 222925 (37.2%) | 24927 (38.4%) | 51457 (37.6%) | 36826 (34.1%) | 40343 (37.0%) | 27525 (39.6%) | 41847 (37.9%) |
| **Divorced** | 98465 (16.4%) | 9252 (14.3%) | 22363 (16.3%) | 21313 (19.7%) | 17798 (16.3%) | 9737 (14.0%) | 18002 (16.3%) |
| **Educational attainment** |  |  |  |  |  |  |  |
| **Higher secondary education** | 221570 (37.8%) | 25026 (38.9%) | 49171 (36.5%) | 45147 (43.2%) | 38757 (36.3%) | 23347 (34.2%) | 40122 (37.2%) |
| **No formal or elementary education** | 233406 (39.8%) | 27542 (42.8%) | 58772 (43.6%) | 26690 (25.5%) | 44637 (41.8%) | 31673 (46.4%) | 44092 (40.9%) |
| **Lower secondary education** | 52158 (8.9%) | 4544 (7.1%) | 11118 (8.3%) | 11411 (10.9%) | 9928 (9.3%) | 5701 (8.4%) | 9456 (8.8%) |
| **Higher education** | 79315 (13.5%) | 7232 (11.2%) | 15700 (11.7%) | 21286 (20.4%) | 13530 (12.7%) | 7470 (11.0%) | 14097 (13.1%) |
| **Residing in urban area** |  |  |  |  |  |  |  |
| **No** | 66444 (11.1%) | 11707 (18.0%) | 18435 (13.5%) | 4959 (4.6%) | 12690 (11.6%) | 8418 (12.1%) | 10235 (9.3%) |
| **Residing in urban area** | 532435 (88.9%) | 53219 (82.0%) | 118556 (86.5%) | 103033 (95.4%) | 96475 (88.4%) | 61064 (87.9%) | 100088 (90.7%) |
| **Country of birth** |  |  |  |  |  |  |  |
| **Born in Sweden** | 529250 (88.4%) | 61171 (94.2%) | 122664 (89.5%) | 87825 (81.3%) | 96154 (88.1%) | 63786 (91.8%) | 97650 (88.5%) |
| **Born outside Sweden** | 69630 (11.6%) | 3755 (5.8%) | 14327 (10.5%) | 20167 (18.7%) | 13011 (11.9%) | 5697 (8.2%) | 12673 (11.5%) |
| **Place of death within a specialised palliative care facility** |  |  |  |  |  |  |  |
| **No** | 532610 (88.9%) | 59641 (91.9%) | 125342 (91.5%) | 85606 (79.3%) | 100646 (92.2%) | 63233 (91.0%) | 98142 (89.0%) |
| **Yes** | 66270 (11.1%) | 5285 (8.1%) | 11649 (8.5%) | 22386 (20.7%) | 8519 (7.8%) | 6250 (9.0%) | 12181 (11.0%) |
| **Palliative care diagnosis; ICD-code Z51.5** |  |  |  |  |  |  |  |
| **No** | 539010 (90.0%) | 59470 (91.6%) | 127636 (93.2%) | 87602 (81.1%) | 100736 (92.3%) | 60491 (87.1%) | 103075 (93.4%) |
| **Yes** | 59870 (10.0%) | 5456 (8.4%) | 9355 (6.8%) | 20390 (18.9%) | 8429 (7.7%) | 8992 (12.9%) | 7248 (6.6%) |
| For categorical variables n (%) is presented. For continuous variables Mean (SD) / Median (Min; Max) / n= is presented.  Red colour= > 2% difference  Yellow colour= >1 % difference  No colour= < 1% difference | | | | | | | |

Supplementary Table III. Multivariable logistic regression analysis for residing at home and dying in hospital vs. dying at home (whole population)

|  | | | | | | | Multivariable** | | |
| --- | --- | --- | --- | --- | --- | --- | --- | --- | --- |
| Model | Variable | n | n missing | Value | n (%) of event | OR (95%CI) Place of Death | | p-value |  |
| Final model with year of death as categorical variable and adjusted* for other variables | Year of Death | 319638 | 0 | 2013 vs 2013 | 30138 (67.7%) | 1.00 | | <0.0001*** |  |
|  |  |  |  | 2014 vs 2013 | 29939 (67.6%) | 1.01 (0.98-1.04) | | 0.74 |  |
|  |  |  |  | 2015 vs 2013 | 30454 (66.7%) | 0.96 (0.93-0.99) | | 0.013 |  |
|  |  |  |  | 2016 vs 2013 | 30703 (66.1%) | 0.96 (0.93-1.00) | | 0.039 |  |
|  |  |  |  | 2017 vs 2013 | 30778 (65.8%) | 0.96 (0.92-1.00) | | 0.046 |  |
|  |  |  |  | 2018 vs 2013 | 30519 (65.3%) | 0.94 (0.90-0.98) | | 0.0055 |  |
|  |  |  |  | 2019 vs 2013 | 29113 (64.3%) | 0.90 (0.86-0.94) | | <0.0001 |  |
| Final model with year of death as a continuous variable and adjusted* for other variables | Year of Death continuous | 319638 | 0 | 2013-<2015 | 60077 (67.7%) |  | |  |  |
|  |  |  |  | 2015-<2018 | 91935 (66.2%) |  | |  |  |
|  |  |  |  | 2018-2019 | 59632 (64.8%) | 0.98 (0.97-0.99) | | <0.0001 |  |
| P-values, OR and Area under ROC-curve are based on original values and not on stratified groups. OR is the ratio for the odds for an increase of the predictor of one unit. *Adjusted for sex, age, cause of death, living conditions, single person household, palliative care needs, palliative care diagnosis, marital status, education, residing in urban area, birth country, cared for in specialised palliative care service,Swedish health care regions and number of Hospital beds Area under ROC-curve with 95% CI for multivariable model = 0.66 (0.66-0.66). ***p-value for the entire effect/factor/variable. | | | | | | | | | |

Supplementary Table IV. Multivariable logistic regression analysis for residing in a nursing home and dying in hospital vs. dying in a nursing home for those aged 60 years old and over (whole population).

|  | | | | | | Multivariable** | |
| --- | --- | --- | --- | --- | --- | --- | --- |
| Model | Variable | n | n missing | Value | n (%) of event | OR (95%CI) Place of Death | p-value |
| Final model with year of death as a categorical variable and adjusted* for other variables | Year of Death | 105522 | 0 | 2013 vs 2013 | 2706 (19.5%) | 1.00 | 0.016*** |
|  |  |  |  | 2014 vs 2013 | 2636 (18.9%) | 0.96 (0.89-1.02) | 0.19 |
|  |  |  |  | 2015 vs 2013 | 2755 (18.5%) | 0.94 (0.88-1.01) | 0.090 |
|  |  |  |  | 2016 vs 2013 | 2856 (18.3%) | 0.98 (0.90-1.07) | 0.69 |
|  |  |  |  | 2017 vs 2013 | 2994 (18.1%) | 0.96 (0.87-1.06) | 0.45 |
|  |  |  |  | 2018 vs 2013 | 2680 (16.9%) | 0.88 (0.79-0.97) | 0.011 |
|  |  |  |  | 2019 vs 2013 | 2544 (17.1%) | 0.91 (0.81-1.01) | 0.072 |
| Final model with year of death as a continuous variable and adjusted* for other variables | Year of Death continuous | 105522 | 0 | 2013-<2015 | 5342 (19.2%) |  |  |
|  |  |  |  | 2015-<2018 | 8605 (18.3%) |  |  |
|  |  |  |  | 2018-2019 | 5224 (17.0%) | 0.98 (0.96-1.00) | 0.017 |
| P-values, OR and Area under ROC-curve are based on original values and not on stratified groups. OR is the ratio for the odds for an increase of the predictor of one unit. *Adjusted for sex, age, cause of death, living conditions, single person household, palliative care needs, palliative care diagnosis, marital status, education, residing in urban area, birth country, cared for in specialised palliative care service,Swedish health care regions and number of Hospital beds Area under ROC-curve with 95% CI for multivariable model = 0.77 (0.77-0.77). ***p-value for the entire effect/factor/variable. | | | | | | | |

Supplementary Table V. Multivariable logistic regression analysis for residing at home and dying in a nursing home versus dying at home (whole population)

|  | | | | | | | Multivariable** | | |
| --- | --- | --- | --- | --- | --- | --- | --- | --- | --- |
| Model | Variable | n | n missing | Value | n (%) of event | OR (95%CI) Place of Death | | p-value |  |
| Final model with year of death as a categorical variable and adjusted* for other variables | Year of Death | 224112 | 0 | 2013 vs 2013 | 16467 (53.4%) | 1.00 | | <0.0001*** |  |
|  |  |  |  | 2014 vs 2013 | 16312 (53.2%) | 1.01 (0.98-1.05) | | 0.51 |  |
|  |  |  |  | 2015 vs 2013 | 16778 (52.5%) | 0.97 (0.93-1.01) | | 0.096 |  |
|  |  |  |  | 2016 vs 2013 | 16184 (50.7%) | 0.88 (0.83-0.92) | | <0.0001 |  |
|  |  |  |  | 2017 vs 2013 | 16805 (51.2%) | 0.91 (0.86-0.96) | | 0.0004 |  |
|  |  |  |  | 2018 vs 2013 | 17133 (51.4%) | 0.90 (0.85-0.95) | | 0.0003 |  |
|  |  |  |  | 2019 vs 2013 | 16439 (50.5%) | 0.86 (0.81-0.91) | | <0.0001 |  |
| Final model with year of death as a continuous and adjusted* for other variables | Year of Death continuous | 224112 | 0 | 2013-<2015 | 32779 (53.3%) |  | |  |  |
|  |  |  |  | 2015-<2018 | 49767 (51.5%) |  | |  |  |
|  |  |  |  | 2018-2019 | 33572 (50.9%) | 0.98 (0.97-0.99) | | <0.0001 |  |
| P-values, OR and Area under ROC-curve are based on original values and not on stratified groups. OR is the ratio for the odds for an increase of the predictor of one unit. *Adjusted for sex, age, cause of death, living conditions, single person household, palliative care needs, palliative care diagnosis, year of death, marital status, education, residing in urban area, birth country, cared for in specialised palliative care service,Swedish health care regions and number of Hospital beds Area under ROC-curve with 95% CI for multivariable model = 0.79 (0.79-0.79). ***p-value for the entire effect/factor/variable. | | | | | | | | | |

Supplementary Table VI. Odds ratios from multivariable logistic regression analyses for trends in place of death of people with potential palliative care needs

|  |  | Dying in hospital vs. dying  at home  Home dwellers | | Dying in hospital vs. dying in  nursing home  Nursing home residents  >60 years old | |  |
| --- | --- | --- | --- | --- | --- | --- |
|  | Year of death | OR (95% C.I) | P-Value | OR (95% C.I) | P-Value |  |
| Whole population | 2014 vs 2013 | 0.99 (0.96-1.03) | 0.65 | 0.92 (0.85-1.00) | 0.046 |  |
|  | 2015 vs 2013 | 0.94 (0.91-0.97) | <.001 | 0.96 (0.89-1.04) | 0.35 |  |
|  | 2016 vs 2013 | 0.92 (0.89-0.95) | <.0001 | 0.95 (0.88-1.03) | 0.24 |  |
|  | 2017 vs 2013 | 0.90 (0.87-0.94) | <.0001 | 0.92 (0.85-0.99) | 0.034 |  |
|  | 2018 vs 2013 | 0.90 (0.87-0.93) | <.0001 | 0.85 (0.78-0.92) | <.001 |  |
|  | 2019 vs 2013 | 0.85 (0.83-0.88) | <.0001 | 0.88 (0.81-0.96) | 0.003 |  |
| North region | 2014 vs 2013 | 1.07 (0.97-1.19) | 0.18 | 0.86 (0.66-1.13) | 0.27 |  |
|  | 2015 vs 2013 | 0.97 (0.88-1.08) | 0.62 | 1.18 (0.92-1.52) | 0.20 |  |
|  | 2016 vs 2013 | 0.93 (0.84-1.03) | 0.16 | 1.11 (0.86-1.43) | 0.42 |  |
|  | 2017 vs 2013 | 0.97 (0.87-1.07) | 0.55 | 1.35 (1.06-1.72) | 0.017 |  |
|  | 2018 vs 2013 | 0.87 (0.78-0.96) | 0.008 | 1.05 (0.81-1.35) | 0.72 |  |
|  | 2019 vs 2013 | 0.92 (0.82-1.02) | 0.099 | 0.99 (0.76-1.29) | 0.95 |  |
| Uppsala-Örebro region | 2014 vs 2013 | 1.02 (0.95-1.09) | 0.65 | 1.06 (0.90-1.24) | 0.49 |  |
|  | 2015 vs 2013 | 0.98 (0.92-1.05) | 0.64 | 0.92 (0.78-1.07) | 0.28 |  |
|  | 2016 vs 2013 | 0.91 (0.85-0.97) | 0.006 | 0.95 (0.81-1.11) | 0.49 |  |
|  | 2017 vs 2013 | 0.91 (0.85-0.97) | 0.005 | 0.95 (0.81-1.11) | 0.51 |  |
|  | 2018 vs 2013 | 0.88 (0.83-0.95) | <.001 | 0.79 (0.67-0.92) | 0.003 |  |
|  | 2019 vs 2013 | 0.83 (0.77-0.88) | <.0001 | 0.77 (0.66-0.91) | 0.002 |  |
| Stockholm region | 2014 vs 2013 | 1.01 (0.93-1.09) | 0.80 | 1.13 (0.93-1.38) | 0.21 |  |
|  | 2015 vs 2013 | 0.96 (0.89-1.04) | 0.37 | 1.12 (0.92-1.36) | 0.26 |  |
|  | 2016 vs 2013 | 0.93 (0.86-1.01) | 0.076 | 1.11 (0.92-1.34) | 0.30 |  |
|  | 2017 vs 2013 | 1.01 (0.93-1.09) | 0.87 | 1.04 (0.86-1.26) | 0.68 |  |
|  | 2018 vs 2013 | 1.09 (1.01-1.18) | 0.028 | 0.96 (0.79-1.17) | 0.70 |  |
|  | 2019 vs 2013 | 1.05 (0.97-1.13) | 0.28 | 1.16 (0.95-1.41) | 0.14 |  |
| West region | 2014 vs 2013 | 1.02 (0.94-1.10) | 0.71 | 0.84 (0.70-1.00) | 0.050 |  |
|  | 2015 vs 2013 | 0.98 (0.91-1.06) | 0.63 | 0.97 (0.82-1.15) | 0.73 |  |
|  | 2016 vs 2013 | 0.92 (0.86-1.00) | 0.045 | 0.89 (0.75-1.06) | 0.19 |  |
|  | 2017 vs 2013 | 0.85 (0.79-0.92) | <.0001 | 0.82 (0.69-0.97) | 0.021 |  |
|  | 2018 vs 2013 | 0.88 (0.82-0.96) | 0.002 | 0.92 (0.78-1.09) | 0.32 |  |
|  | 2019 vs 2013 | 0.83 (0.77-0.90) | <.0001 | 0.82 (0.68-0.98) | 0.026 |  |
| Southeast region | 2014 vs 2013 | 1.00 (0.91-1.09) | 0.93 | 0.94 (0.75-1.18) | 0.60 |  |
|  | 2015 vs 2013 | 0.98 (0.89-1.07) | 0.64 | 0.91 (0.72-1.14) | 0.39 |  |
|  | 2016 vs 2013 | 0.95 (0.87-1.05) | 0.33 | 0.88 (0.70-1.11) | 0.27 |  |
|  | 2017 vs 2013 | 0.89 (0.81-0.97) | 0.011 | 0.71 (0.56-0.89) | 0.003 |  |
|  | 2018 vs 2013 | 0.89 (0.81-0.98) | 0.015 | 0.82 (0.65-1.03) | 0.094 |  |
|  | 2019 vs 2013 | 0.80 (0.73-0.88) | <.0001 | 0.92 (0.73-1.16) | 0.49 |  |
| South region | 2014 vs 2013 | 0.85 (0.79-0.92) | <.0001 | 0.74 (0.62-0.88) | <.001 |  |
|  | 2015 vs 2013 | 0.77 (0.71-0.83) | <.0001 | 0.75 (0.63-0.88) | <.001 |  |
|  | 2016 vs 2013 | 0.87 (0.81-0.93) | <.001 | 0.82 (0.69-0.96) | 0.016 |  |
|  | 2017 vs 2013 | 0.82 (0.76-0.88) | <.0001 | 0.78 (0.66-0.91) | 0.002 |  |
|  | 2018 vs 2013 | 0.79 (0.74-0.85) | <.0001 | 0.64 (0.54-0.75) | <.0001 |  |
|  | 2019 vs 2013 | 0.74 (0.69-0.80) | <.0001 | 0.70 (0.58-0.83) | <.0001 |  |
| P-values, Odds ratios (OR) and Area Under ROC are based on original values and not on stratified groups.  Area under ROC-curve with 95% CI for multivariable models range from 0.60-0.79 | | | | | | |


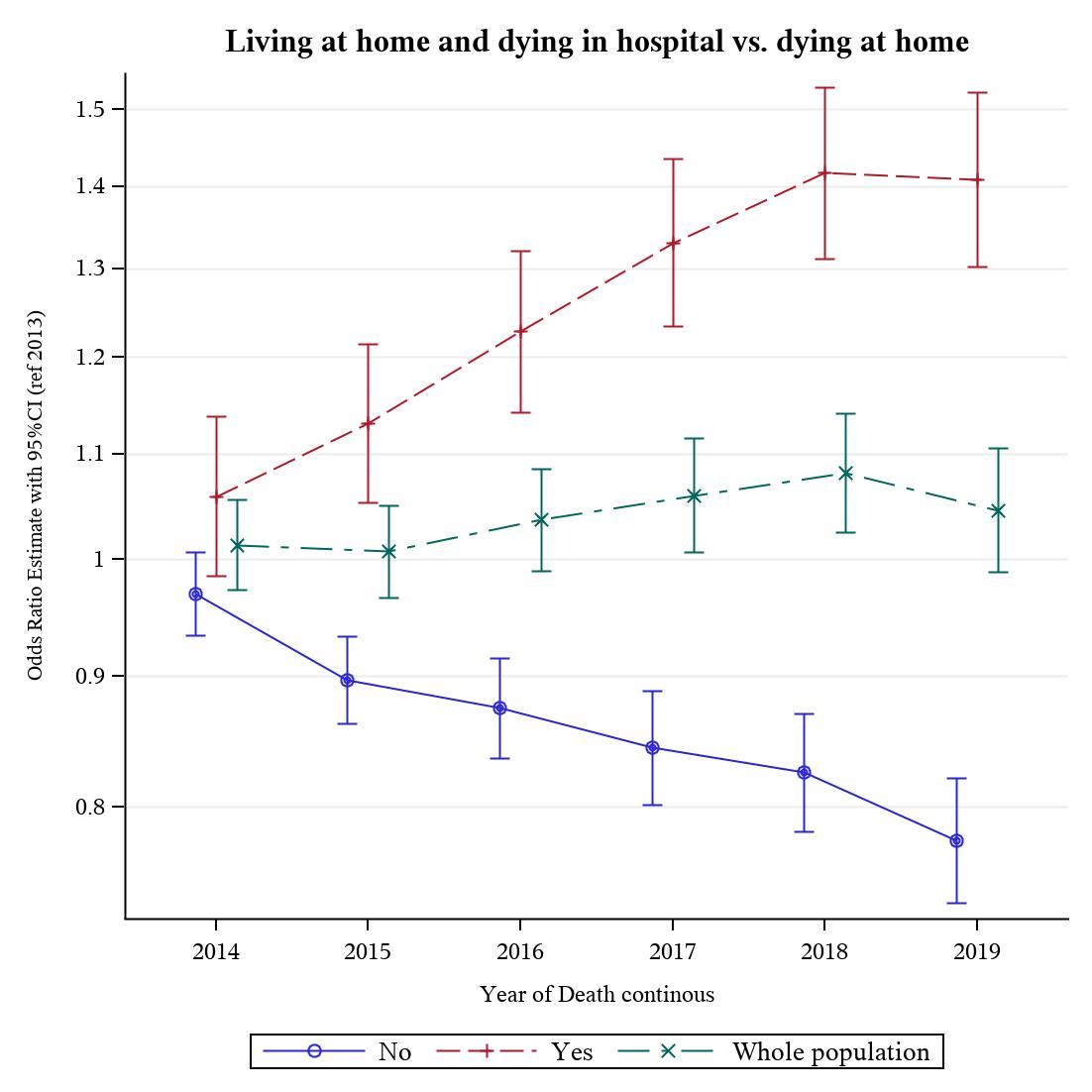


Supplementary Figure I. Interaction of year of death with having received specialised palliative care during the last week of life for those living at home.

*Curves are comparisons with 2013 for each year from 2014 to 2019.


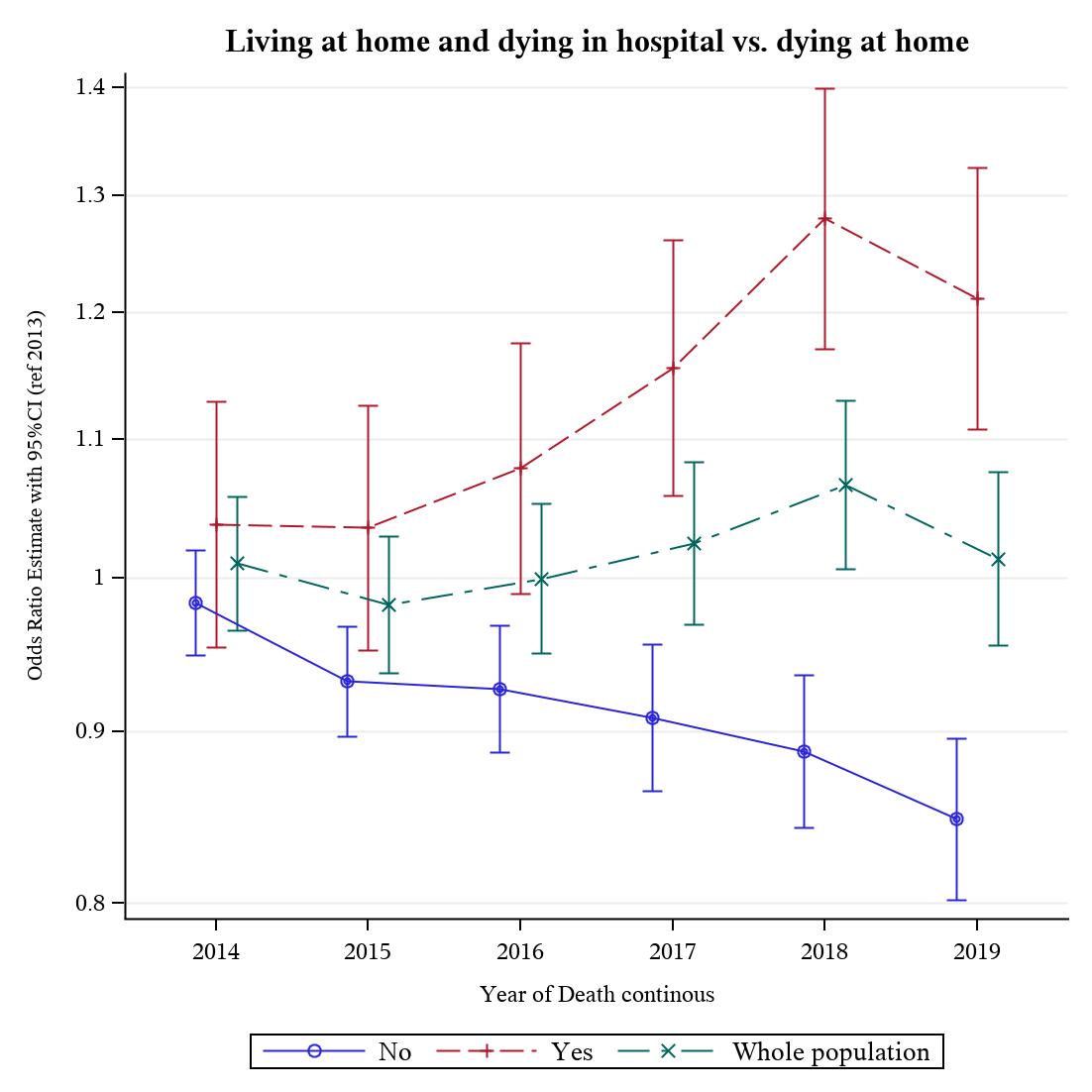


Supplementary Figure II. Interaction of year of death with having been diagnosed with ICD-10 code Z51.5 for palliative care for those living at home.

*Curves are comparisons with 2013 for each year from 2014 to 2019.


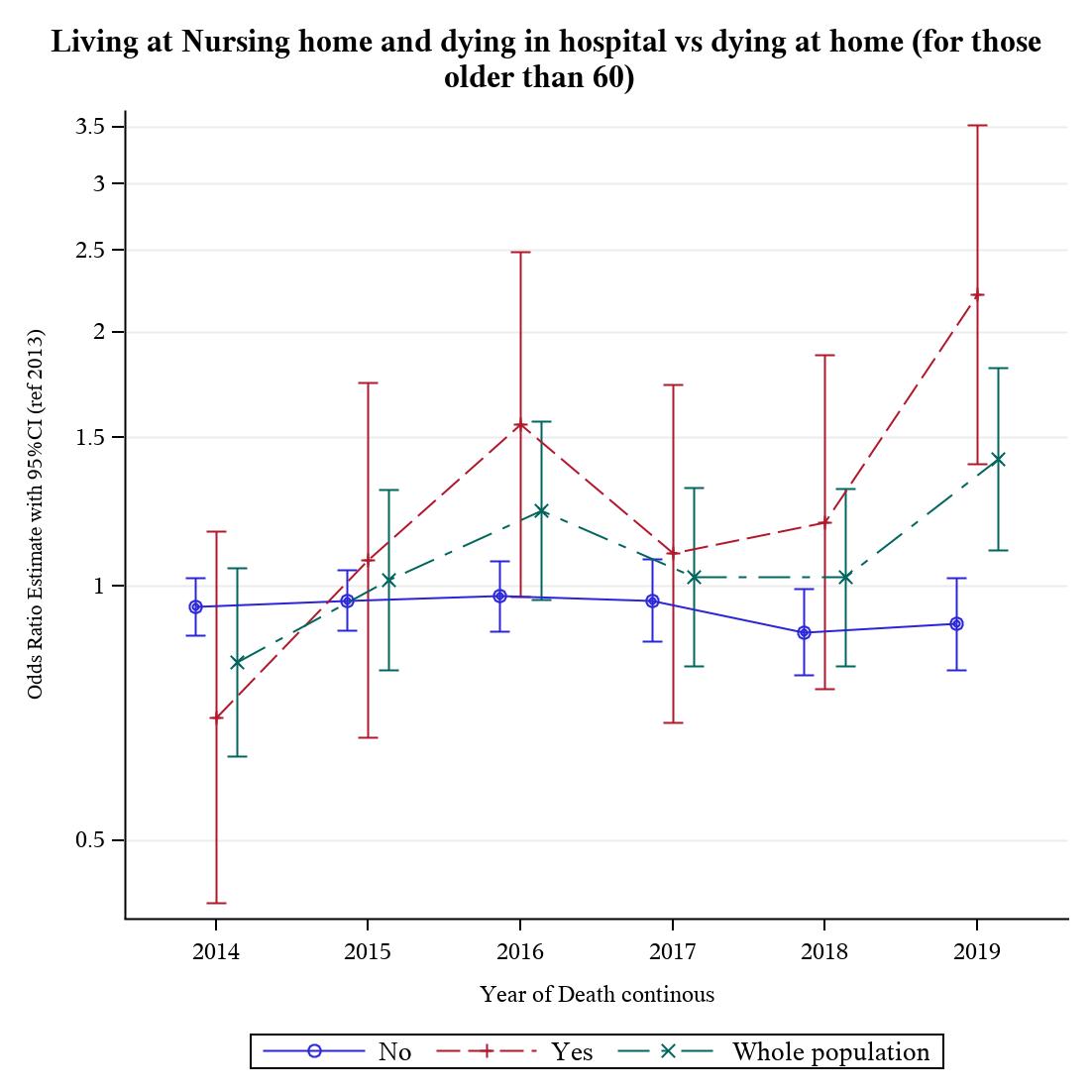


Supplementary Figure III. Interaction of year of death with having been diagnosed with ICD-10 code Z51.5 for palliative care for those living in a nursing home.

*Curves are comparisons with 2013 for each year from 2014 to 2019.
